# Clinical Experience Using a 3D-Printed Patient-Specific Instrument for Medial Opening Wedge High Tibial Osteotomy

**DOI:** 10.1155/2018/9246529

**Published:** 2018-05-08

**Authors:** Jesse Chieh-Szu Yang, Cheng-Fong Chen, Chu-An Luo, Ming-Chau Chang, Oscar K. Lee, Ye Huang, Shang-Chih Lin

**Affiliations:** ^1^Department of Orthopedic & Traumatology, Taipei Veterans General Hospital, Taipei, Taiwan; ^2^Institute of Clinical Medicine, National Yang-Ming University, Taipei, Taiwan; ^3^Department of Orthopedics, School of Medicine, National Yang-Ming University, Taipei, Taiwan; ^4^Graduate Institute of Biomedical Engineering, National Taiwan University of Science and Technology, Taipei, Taiwan; ^5^Institute of Anatomy and Cell Biology, National Yang-Ming University, Taipei, Taiwan; ^6^Department of Orthopedic Surgery, Taipei City Hospital and Institute of Clinical Medicine, National Yang-Ming University, Taipei, Taiwan; ^7^Adult Orthopedic Department, Beijing JST Hospital, Beijing, China

## Abstract

**Purpose:**

High tibial osteotomy (HTO) has been adopted as an effective surgery for medial degeneration of the osteoarthritis (OA) knee. However, satisfactory outcomes necessitate the precise creation and distraction of osteotomized wedges and the use of intraoperative X-ray images to continually monitor the wedge-related manipulation. Thus HTO is highly technique-demanding and has a high radiation exposure. We report a patient-specific instrument (PSI) guide for the precise creation and distraction of HTO wedge.

**Methods:**

This study first parameterized five HTO procedures to serve as a design rationale for an innovative PSI guide. Preoperative X-ray and computed tomography- (CT-) scanning images were used to design and fabricate PSI guides for clinical use. The weight-bearing line (WBL) of the ten patients was shifted to the Fujisawa's point and instrumented using the TomoFix system. The radiological results of the PSI-guided HTO surgery were evaluated by the WBL percentage and tibial slope.

**Results:**

All patients consistently showed an increased range of motion and a decrease in pain and discomfort at about three-month follow-up. This study demonstrates the satisfactory accuracy of the WBL adjustment and tibial slope maintenance after HTO with PSI guide. For all patients, the average pre- and postoperative WBL are, respectively, 14.2% and 60.2%, while the tibial slopes are 9.9 and 10.1 degrees. The standard deviations are 2.78 and 0.36, respectively, in postoperative WBL and tibial slope. The relative errors of the pre- and postoperative WBL percentage and tibial slope averaged 4.9% and 4.1%, respectively.

**Conclusion:**

Instead of using navigator systems, this study integrated 2D and 3D preoperative planning to create a PSI guide that could most likely render the outcomes close to the planning. The PSI guide is a precise procedure that is time-saving, radiation-reducing, and relatively easy to use. Precise osteotomy and good short-term results were achieved with the PSI guide.

## 1. Introduction

Medial opening wedge high tibial osteotomy (HTO) has been used to treat osteoarthritic knees with medial cartilage degeneration, especially for young and active patients [1–3]. This procedure is more favorable than lateral closing wedge HTO, which often induces complications such as compartmental syndrome, lateral muscle detachment, proximal fibula osteotomy, and limb shortening, as well as neurological complications [4–6]. Several studies have shown satisfactory outcomes with medial opening wedge HTO. Floerkemeier et al. reported favorable midterm results after using medial opening wedge HTO, even in older patients with a great deal of cartilage damage [7]. Bode et al. and Harris et al. reported over 96% and 92.4% survival rates, respectively, with excellent clinical outcomes five years after medial opening wedge HTO [8, 9]. Approximately 90% of patients returned to work or their sport within one year in Ekhtiari's follow-up study [10]. Patients in a study by Duivenvoorden et al. had a survival rate of up to 90% ten years after medial opening wedge HTO [11].

To achieve a satisfactory outcome when performing medial opening wedge HTO, patient selection and surgical technique are important factors [12–14]. In practice, manual creation and distraction of HTO wedges are highly technique-demanding, especially for an inexperienced surgeon [7]. During the surgical procedure, potential complications, such as fractures of the lateral cortex or tibial plateau, dislocation of the lateral hinge, change in the tibial slope, and over- or undercorrection can adversely affect clinical outcomes [15]. Therefore, both accurate preoperative planning and precise intraoperative execution are necessary to obtain a satisfactory outcome with medial opening wedge HTO [16].

**Figure 1:**
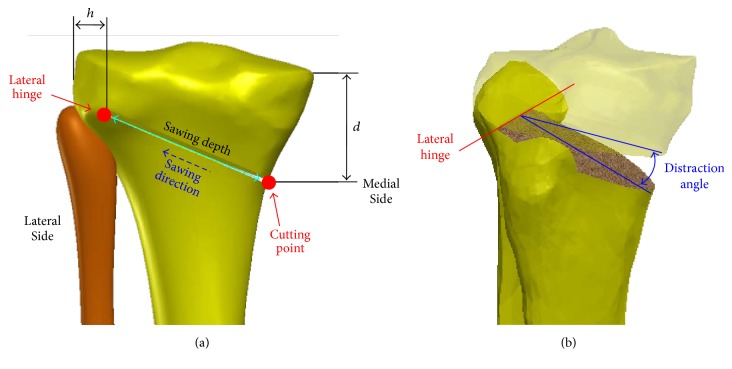
Schematic diagrams of surgical parameters for HTO with a medial opening wedge. (a) Cutting position, lateral hinge, sawing direction, and sawing depth in the coronal plane. (b) 3D diagram of the lateral hinge and distraction angle. The symbols are defined in the content.

There are five parameters of medial opening wedge HTO, including cutting point, lateral hinge, sawing direction, sawing depth, and correction angle (Figure 1). The first is the entry position of the osteotomized wedge, which is associated with the distance *d* between the cutting point and the medial tibial plateau (Figure 1(a)). As the second parameter, the lateral hinge is considered to be a rotation axis in the valgus correction procedure. It is suggested to be about 5-mm (*h*) from the lateral border of the tibia plateau (Figure 1(a)) and should be located at the upper border of the proximal tibiofibular joint [15, 17].

In theory, the 3D variation of the lateral hinge can alter the opening height under the same correction angle and thus lead to an undesired change of the tibial slope [15, 18, 19]. The sawing direction and depth are determined by the orientation and distance between the cutting point and the lateral hinge, respectively. In the situation of excessive sawing depth, the wedge distraction potentially puts the lateral cortex at risk of fracture due to deficient remaining bone (i.e., the hinge is located too laterally) [20]. However, lack of sawing depth potentially leads to the intracondylar fracture of the proximal tibia [15]. Correction angle is the last parameter that directly affects the final HTO result [21]. Based on lower limb radiographs, the determination of the correction angle depends on the target zone for the weight-bearing line (WBL), which is defined as the line connecting the centers of the hip and ankle joints [3, 14]. Excessive or insufficient correction intraoperatively cannot adjust the loading ratio of the medial and lateral sites, as in the preoperative planning [22].

The aforementioned HTO parameters can be measured and confirmed intraoperatively by commonly used fluoroscopy methods [23]. However, surgical accuracy with the use of these methods is likely to suffer due to technique error resulting from manual and visual factors [24]. Computer-navigated techniques and novel instrumentation have been introduced recently to improve HTO accuracy [24–26]. Patient-specific instruments (PSI) that have been used in the field of hip and knee arthroplasty can serve as an alternative [27, 28]. The PSI rationale is to create a guiding instrument for the surfaces that can fit well with the targeting region of the patient's bone. The benefits include increased accuracy, decreased surgery time, and elimination of the need for extra devices or reference markers. This study aims to report on a novel PSI guide (designed by a company specializing in the production of orthopedic implants, A Plus Biotechnology Co. Ltd., New Taipei City, Taiwan) for precise osteotomy and accurate distraction of medial opening wedge HTO. The design parameters and clinical outcomes of PSI-based HTO are described and discussed. Two comparison indices of ten patients are evaluated in this clinical study. We hypothesize that the PSI provides an accurate and repeatable method for adjusting the WBL of medial OA patients.

## 2. Materials and Methods

### 2.1. Patient Information

Ten patients (4 males and 6 females) voluntarily enrolled in this study. The average age was 67.2 years, ranging from 56 to 79 years. All patients were diagnosed as medial OA knee with varus deformity. The diagnostic MRI scans revealed significant degeneration of the medial articular cartilage of the medial femoral condyle; the articular cartilage of the lateral femoral condyle was intact. The patients uniformly decided to use the PSI guide after they had been informed about the entire surgical procedure, so as to avoid total knee replacement. The Medical Ethics Committee of the National Taiwan University of Science and Technology approved the study design. Written informed consent for participation in the study and publication of the accompanying images was obtained from all patients.

### 2.2. 2D Preoperative Planning

**Figure 2:**
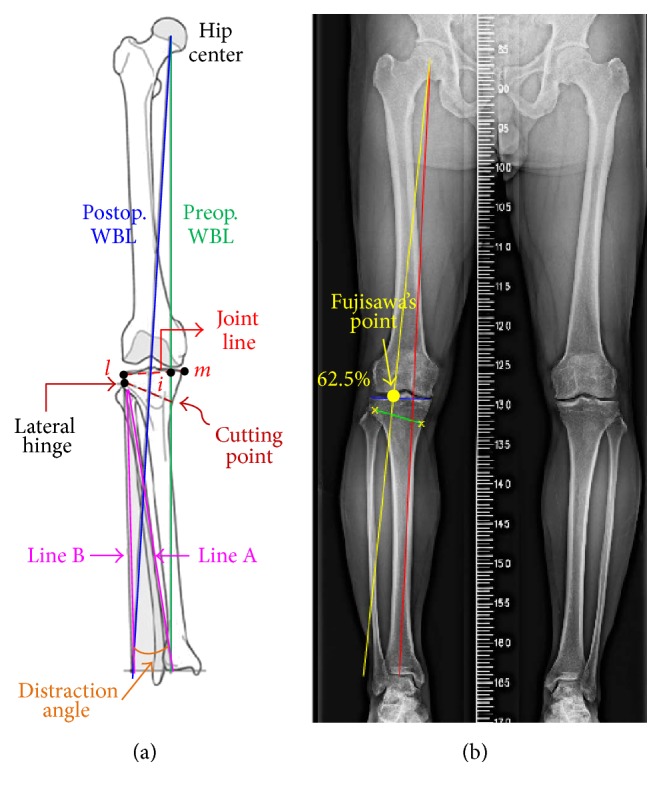
(a) Schematic diagram of the 2D preoperative planning for WBL percentage and distraction angle. (b) Fujisawa's point is adopted as a preplanning guide in the limb radiograph.

The correction angle was determined by preoperative weight-bearing coronal plane radiography of the entire leg (Figure 2). The WBL of the lower limb was measured initially and the intersection point (*i*) between the WBL and the joint line was determined as a preoperative index of the WBL percentage (Figure 2(a)) [3, 14]. The joint line was directed from the medial (point *m*) to the lateral (point *l*) edges of the tibial plateau. The WBL percentage was defined as the width ratio of the medial to the intersection point (line *mi*) and the tibial plateau (line *ml*). Around the hip center, the postoperative WBL was then simulated by rotating the preoperative WBL until it passed through the Fujisawa's point (Figure 2(b)), which suggested 62.5% of the WBL percentage [14]. Approximately 35 mm below the joint line, the most concave point on the proximal medial tibial border was set to be the cutting point (Figure 2(a)). The lateral hinge was assumed to be orthogonal to the coronal plane and located at the upper border of the tibiofibular joint [17]. Around the lateral hinge, the postoperative WBL was determined to pass through the Fujisawa's point, thus the correction angle was equal to the angle between the lines A and B (Figure 2(a)).

Using Matlab software (MathWorks Inc., Natick, MA, USA), the anatomical characteristics and surgical parameters, including the hip center, ankle center, joint line, cutting point, and lateral hinge, were identified to calculate the correction angle for the desired postoperative WBL (Figure 2(b)). In this case, the correction angle of the right knee was 8.2 degrees when the postoperative WBL passed 62.5% of the tibial width.

### 2.3. 3D Preoperative Planning

**Figure 3:**
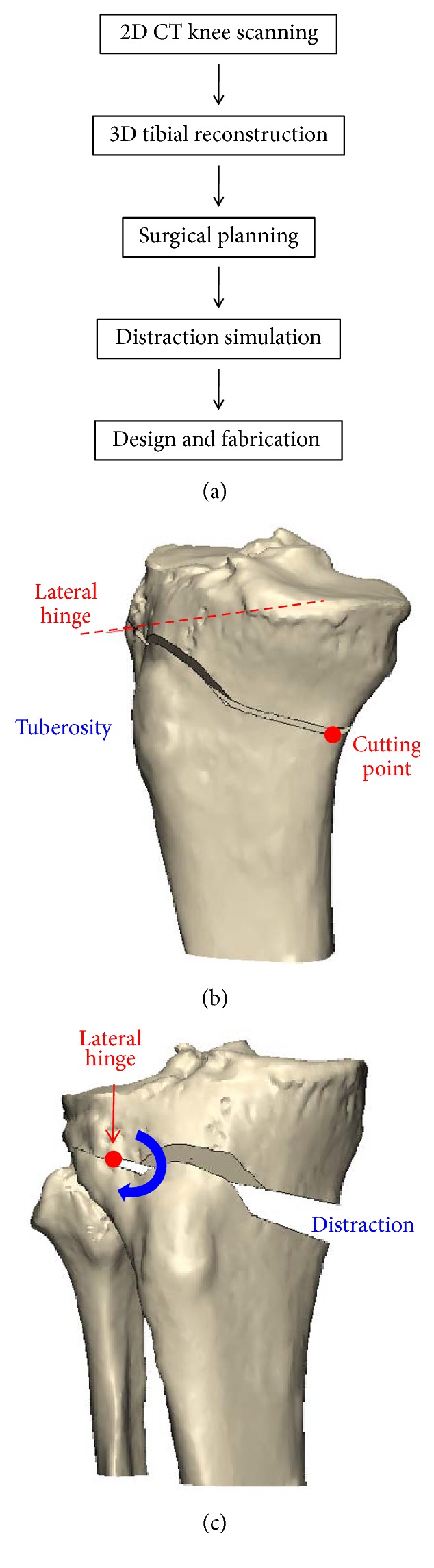
(a) Flow chart of the preoperative planning strategy. (b) Simulation of the biplanar cut. (c) Valgus correction of the 3D tibial model.

The 3D preoperative HTO planning can be divided into six steps (Figure 3(a)). The CT-scanning images of the knee joint were executed with a 1-mm slice separation. The tibial 3D model was reconstructed with triangular surface meshes using Amira 4.0 software (Mercury Computer Systems, Inc., Berlin, Germany). The surface model of the proximal tibia was further transformed into a solid model with smooth and seamless surfaces using SolidWorks Ed. 2015 software (SolidWorks Corporation, Concord, MA, USA). The coronal plane of the reconstructed tibia was created and aligned with the full-leg radiographs that were used for the 2D preoperative planning. With this, the cutting point and lateral hinge could be identified again for the 3D planning, and the sawing direction could further be defined.

The strategy of biplanar osteotomy [17] was adopted and simulated by cutting obliquely from the cutting point to the lateral hinge in the manner of an ascending anterior cut to preserve tibial tuberosity (Figure 3(b)). Next, the distal portion of the osteotomized tibia was valgus rotated with respect to the lateral hinge (Figure 3(c)). From the trigonometric perspective, the rotation angle was equal to the correction angle that was derived from the 2D preoperative planning. To maintain the tibial slope, the original and osteotomized tibiae model were compared at this stage. If the slope was changed, the lateral hinge would have to be adjusted until it achieved original slope.

**Figure 4:**
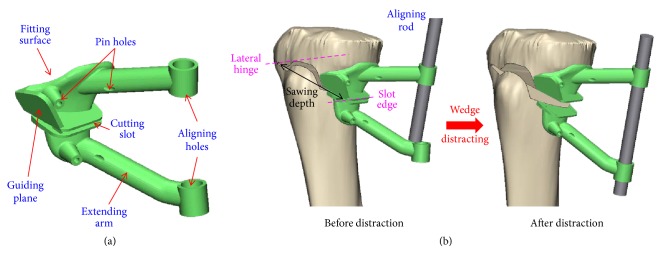
Schematic diagrams of (a) the PSI guide for medial opening wedge HTO and (b) intraoperative usage before and after distraction.

A surgical guide was designed to fit the medial tibial surface with four pin holes to stably fix the guide on the targeting region of the patient's bone. A cutting slot and a guiding plane were provided for the biplanar osteotomy (Figure 4(a)). The edge of the cutting slot was parallel to the lateral hinge, and the sawing depth was defined as specific integer from the slot edge to hinge (Figure 4(b)). The guiding plane coincided with the anterior cutting plane, and thus the oscillating saw could lean against the plane while sawing. For intraoperative confirmation of the correction angle, two extended arms with two holes above and below the osteotomized wedge were, respectively, created (Figure 4(a)). These two holes were designed not to be initially aligned until the wedge was distracted to the correction angle, as in the preoperative planning. At this moment, an aligning rod was used to pass through the two aligned holes, to assist the surgeon in determining that the correction angle was achieved (Figure 4(b)). The PSI guide was then manufactured by a commercial 3D printer (Formlabs, Cambridge, MA) using biocompatible resin (Dental SG, EN-ISO 10993-1:2009/AC:2010, USP Class VI). Before clinical use, moist heat sterilization was adopted and no visible deformation of the PSI profile was allowed to affect the PSI accuracy.

### 2.4. Intraoperative Application

**Figure 5:**
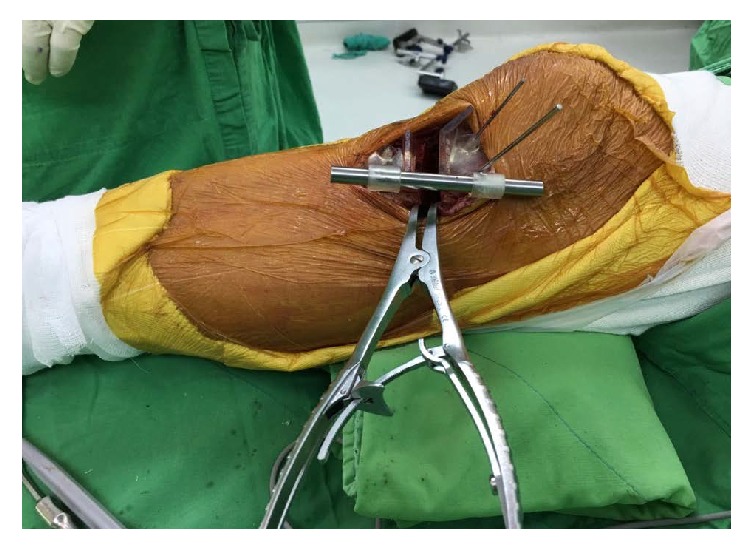
Intraoperative photograph with the PSI guide.

A 4-cm incision was made longitudinally in the proximal and anteromedial portion of the tibia, followed by dissection of the pes anserinus and superficial medial collateral ligament. Then, the PSI guide was attached to the targeting region, as simulated in the virtual models (Figure 4). K-wires were drilled through the holes to fix the guide. Using the cutting slot and guiding plane, a biplanar cut was performed carefully with an oscillating saw in order to create the desired hinge. During the sawing process, the sawing depth was monitored by the length scale of the saw blade. After two wedges had been created, the guide was separated into proximal and distal parts from the cutting slot. For use as an aligning tool, the rod was inserted through the proximal hole, but it was unable to pass through the distal hole at this moment. An osteotome was inserted gradually and carefully until the length scale reached the sawing depth, followed by serial insertion of another osteotome for distraction. The insertion was stopped when the rod was able to cross the proximal and distal holes. A spreader was used to hold the distracted wedge before removing the osteotomes. The aligning mechanism of the PSI holes and aligning rod could significantly simplify the wedge distraction (Figure 5). The distal K-wires were removed before taking the radiographs. Finally, the TomoFix plate (TomoFix, Synthes GmbH, Switzerland) and locking screws were used to fix the osteotomized tibia. The surgical procedure was completed in about 20 minutes.

### 2.5. Postoperative Evaluation

Using radiographs, the postoperative evaluation focused on the transformation of limb geometry and knee biomechanics in terms of both WBL percentage and tibial slope. The weight-bearing radiographs of the full legs at the preoperative period served as a comparison baseline, that the PSI parameters are designed to rearrange WBL percentage and tibial slope. About six weeks postoperatively, similar radiographs were taken and compared with the preoperative counterpart. The shifted WBL percentage and unchanged tibial slope were used to validate the function of the PSI guide.

## 3. Results

**Figure 6:**
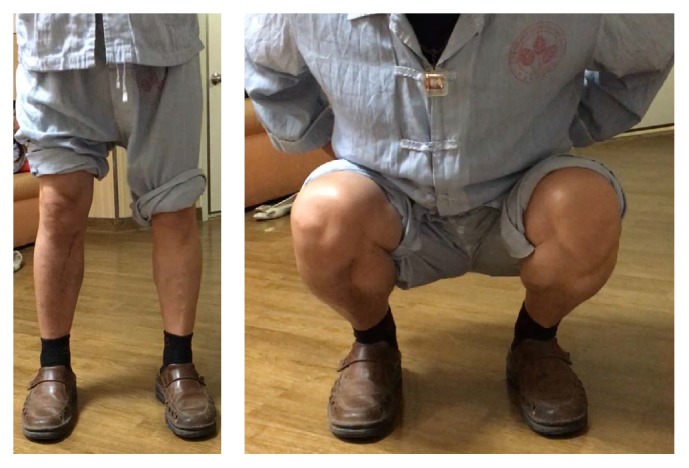
Postoperative photographs of a patient in standing and squatting positions three months after surgery.

The first patient (number 1) was seen in the clinic three months postoperatively (Figure 6). He had good wound healing and a satisfactory range of motion. The patient's range of motion continued to increase during the following three months, to a range of 0–140 degrees. At the six-month follow-up, the patient reported that he had returned to his normal work routine. His knee had a full range of motion, and the swelling in his knee was improved compared to the preoperative situation. He still experienced some pain at the medial joint line of the knee but was fully functional. At his one-year follow-up, the medial joint line pain was no longer present. He still experienced occasional discomfort due to the plate but was very pleased with the outcome.

**Figure 7:**
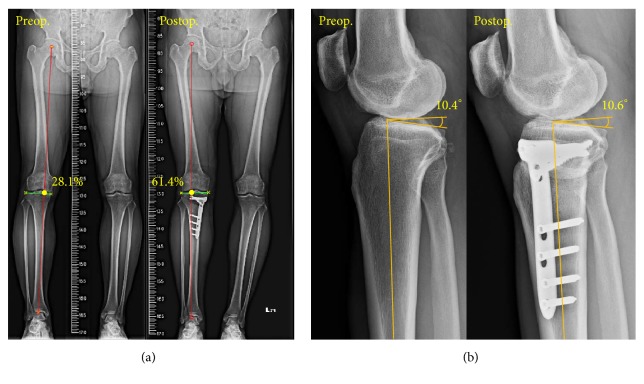
Pre- and postoperative comparisons of (a) the WBL percentage, with the negative value indicating that the WBL passes outside medially of the entire joint, and (b) the tibial slope.

**Figure 8:**
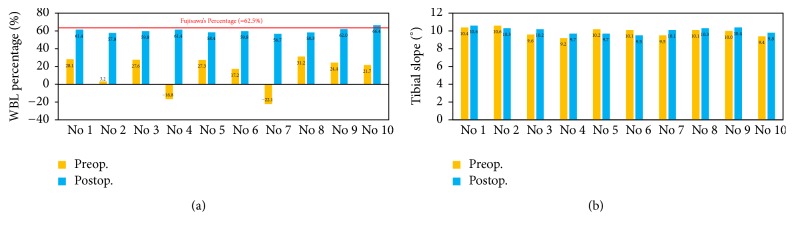
Pre- and postoperative data of ten HTO patients: (a) WBL percentage and (b) tibial slope.

The pre- and postoperative radiographs of two comparison indices for the first patient showed that the WBL percentages were 28.1% and 61.4% before and after surgery (Figure 7). The error between the corrected and target WBL percentage was only 1.76%. The error of the pre- and postoperative tibial slopes was only 1.9% (Figure 7(b)). For all patients, the average pre- and postoperative WBL are, respectively, 14.2% and 60.2%, while the tibial slopes are 9.9 and 10.1 degrees. The standard deviations are 2.78 and 0.36, respectively, in postoperative WBL and tibial slope. The average correction angle is 10.6 degrees.  Figure 8 shows the pre- and postoperative WBL and tibial slope for each patient. The negative values of preoperative WBL in patients numbers 4 and 7 indicate that the mechanical axis is located medially to the medial tibial plateau. The relative errors for pre- and postoperative WBL percentage and tibial slope averaged 4.9% and 4.1%, respectively.

## 4. Discussion

With the improvements in surgical techniques and fixation systems, HTO has become an attractive treatment option for medial compartmental osteoarthritis [29]. However, precision surgery is hard to achieve without navigation systems or well-designed instruments [15], especially for inexperienced surgeons. Imprecision surgery might accompany the complications such as fractures of lateral cortex or tibial plateau, hinge dislocation, change of tibial slope, and over- or undercorrection [15]. Jenny et al. reported that approximately 20% of patients do not have the proper correction using conventional HTO techniques [30]. The tibial plateau fracture rate has been informed to be as high as 11% [2]. Miniaci et al. suggested that only 50% of cases achieved the desired range of correction [31]. These problems can be attributed to inaccurate preoperative planning, an imprecise intraoperative technique, and inherent error while measuring [24]. Moreover, implant failure or wedge nonunion can also be attributed to postoperative loss of correction [32].

In practice, a metallic rod or cable connecting both hip and ankle centers is commonly used as the WBL to verify the intraoperative correction [24]. However, this method still has the potential to contribute to technical error (e.g., due to the use of a rough estimate of the WBL percentage), even though it is commonly used [26]. By comparison, use of the aligning rod provides a more precise method to accomplish the distraction of the desired WBL percentage (Figure 4(b)). This kind of WBL measurement is based on the non-weight-bearing condition, while the preoperative planning and postoperative result are measured under a weight-bearing condition. This will lead to up to a 2-degree difference in wedge distraction [33]. The aligning holes of the PSI guide allow the confirmation of the completion of the desired WBL percentage. However, the conformation is still carried out in a non-weight-bearing condition.

Navigation systems have been employed to increase the surgical accuracy of HTO. Kim et al. reported excellent outcomes using the kinematic navigation system [23]. Heijens et al. reported good intraoperative control of the correction using an HTO navigation device [34]. Reising et al. showed that the use of navigation systems can eliminate the outliers of a defined range [26]. Picardo et al. systematically reviewed clinical and cadaver HTO studies [35] and concluded that the use of a navigation system holds some advantages, but there may be technique pitfalls, such as line-of-sight issues, registration failures, and mechanical or software malfunctions. Besides, learning curve issues with navigation techniques are reported to contribute to a higher complication rate [33]. Even with an experienced operator, additional operating time is still required. Other potential disadvantages include extra equipment demands, higher costs, and WBL measurement without a weight-bearing load [24].

Munier et al. reported a predrilled PSI guide for HTO with satisfactory outcomes [36]. The correction was achieved through the alignment of the predrilled holes and the plate holes. Compared with the current study, the predrilled method allowed a smaller volume of PSI when a short plate is used; thus a minimum incision can be attained. If the opening wedge was fixed with TomoFix system, the predrilled holes might yield larger errors during the procedure of inserting the lag screw, which can provide the compression force and stability for the lateral hinge [17]. Neither the predrilled holes nor our aligning rod works well if the intraoperative-created lateral hinge differs greatly with the preoperative planning. To avoid this situation, the sawing depth is engraved on our PSI, and the edge of cutting slot is designed in parallel with the planned hinge direction. Same as all CT-based PSIs, they are designed based on the reconstructed bone models without the soft tissues. However, the technique of soft tissue release will also affect the tibial slope and correction result [37]. Predrilled holes might cause inconvenience once the operator wants to adjust the amount of correction intraoperatively due to the soft tissue problems.

This study demonstrates the satisfactory outcomes in terms of the WBL shift and tibial slope maintenance with PSI guides for HTO. The average tourniquet time was about 30 minutes, and blood loss was under 20 ml after wound closure. The errors between preoperative planning and surgical results averaged 4.9% and 4.1%, respectively, in WBL percentage and tibial slope (Figure 8). This was attributed to the well-designed PSI guide. The cutting slot with a specified sawing depth can adjust the orientation of the lateral hinge to a position that is close to preoperative planning. As a result, the postoperative tibial slope was nearly equal to the original slope (Figure 8(b)). It is believed that the tibial slope can be adjusted clinically by controlling the anterior and posterior wedge height [4]. In some cases, the tibial slope might tend to decrease or increase in patients with ACL deficiency or PCL injury [3]. In these cases, the orientation of the lateral hinge can be adjusted by means of rearranging the cutting slot of the PSI guide. However, this was not extensively investigated in this study.

Postoperative limb alignment is regarded as an important factor in evaluating the surgical outcome. This PSI guide provides an effective and convenient method to monitor the WBL percentage and avoid using fluoroscopic methods and navigation systems. In turn, operating time and radiologic exposure are reduced. Despite the good outcome when using the PSI guide, there are some inherent issues. The aligning rod is coaxial to the proximal and distal holes only when the other surgical parameters are consistent with the theoretical preplanning. This implies that every step in the osteotomized procedure needs to be precisely performed. Nevertheless, the aligning holes will be mismatched if the lateral cortex fractures or the PSI guide is dislocated. The necessity of CT scanning for PSI design might increase extra radiation exposure to the patient. However, the exposure level of intraoperative fluoroscopy is about 20–200 mSv per minute [38], but only 0.16 mSv for the CT scanning of knee [39]. Furthermore, intraoperative C-arm use might be associated with radiation exposure to the surgeons and assistants. There are some disadvantages to the use of the PSI guide. A larger surgical exposure is required to allow the PSI guide to be attached onto the bone. The preoperative design and manufacture of the PSI guide are time-consuming and more expensive for patients. And, misunderstanding in communication between surgeons and engineers often occurs [40].

Some limitations of this study should be noted. The distraction angle is calculated through the 2D radiograph of the lower limb, while the PSI guide and tibial slope are designed and checked on the 3D tibial model, so errors may occur in the measurement and registration from the 2D to the 3D images. The lateral hinge is theoretically orthogonal to the coronal plane; thus the wedge can be distracted along the coronal plane. However, in order to maintain the original tibial slope, the lateral hinge often has to be slightly adjusted in the 3D manner. The systematic strategy of adjusting the tibial slope was not discussed in this study. Between the steps of removing the PSI guide and plate fixation, a spreader was used to hold the distracted wedge; this might lead to the loss of correction angle. Surgical outcomes might be affected by the measuring of the pre- and postoperative radiographs, since there may be differences in standing posture when the radiographs are taken. This is also not discussed in this study. Although the short-term results reveal satisfactory outcomes with the precise osteotomy and that no fracture occurred, long-term follow-up evaluations of various knee functions are still necessary.

## 5. Conclusion

Even with perfect preoperative planning, the practical difficulty is in transitioning from planning to the actual surgical procedure. Instead of using a navigator system, this study integrated 2D and 3D preoperative planning to create a PSI guide that can most likely yield outcomes that are close to the planning. Compared with the navigation system, the use of the PSI guide prevents the requirement of continuous tracking and registration that potentially increases surgical errors and consumes time. The advantages of the PSI guide are that it is time-saving, radiation-reducing, and relatively easy to use and that it is a precise procedure. The precise osteotomy is performed with the PSI guide, and good short-term results are achieved without the use of a navigation system.
